# Effectiveness of intra-articular lidocaine injection for reduction of anterior shoulder dislocation: randomized clinical trial

**DOI:** 10.1590/S1516-31802012000600003

**Published:** 2013-01-18

**Authors:** Marcel Jun Sugawara Tamaoki, Flavio Faloppa, André Wajnsztejn, Nicola Archetti, Marcelo Hide Matsumoto, João Carlos Belloti

**Affiliations:** I MD, PhD. Attending Physician in the Shoulder and Elbow Sector, Department of Orthopedics and Traumatology, Escola Paulista de Medicina, Universidade Federal de São Paulo (EPM-Unifesp), São Paulo, Brazil.; II MD, PhD. Titular Professor and Head of the Department of Orthopedics and Traumatology, Escola Paulista de Medicina, Universidade Federal de São Paulo (EPM-Unifesp), São Paulo, Brazil.; III MD. Attending Physician in the Traumatology Sector, Department of Orthopedics and Traumatology, Escola Paulista de Medicina, Universidade Federal de São Paulo (EPM-Unifesp), São Paulo, Brazil.; IV MD, PhD. Head of the Shoulder and Elbow Sector, Department of Orthopedics and Traumatology, Escola Paulista de Medicina, Universidade Federal de São Paulo (EPM-Unifesp), São Paulo, Brazil.; V PhD. Adjunct Professor, Department of Orthopedics and Traumatology, Escola Paulista de Medicina, Universidade Federal de São Paulo (EPM-Unifesp), São Paulo, Brazil.

**Keywords:** Shoulder joint, Shoulder dislocation, Emergency medicine, Orthopedics, Traumatology, Articulação do ombro, Luxação do ombro, Medicina de emergência, Ortopedia, Traumatologia

## Abstract

**CONTEXT AND OBJECTIVE:**

Shoulder dislocation is the most common dislocation among the large joints. The aim here was to compare the effectiveness of reduction of acute anterior shoulder dislocation with or without articular anesthesia.

**DESIGN AND SETTING:**

Prospective randomized trial conducted in Escola Paulista de Medicina, Universidade Federal de São Paulo (EPM-Unifesp).

**METHODS:**

From March 2008 to December 2009, 42 patients with shoulder dislocation were recruited. Reductions using traction-countertraction for acute anterior shoulder dislocation with and without lidocaine articular anesthesia were compared. As the primary outcome, pain was assessed through application of a visual analogue scale before reduction, and one and five minutes after the reduction maneuver was performed. Complications were also assessed.

**RESULTS:**

Forty-two patients were included: 20 in the group without analgesia (control group) and 22 in the group that received intra-articular lidocaine injection. The group that received intra-articular lidocaine had a statistically greater decrease in pain over time than shown by the control group, both in the first minute (respectively: mean 2.1 (0 to 5.0), standard deviation, SD 1.3, versus mean 4.9 (2.0 to 7.0, SD 1.5; P < 0.001) and the fifth minute (respectively: mean 1.0; 0 to 3.0; SD = 1.0 versus mean 4.0; 1.0 to 6.0; SD = 1.4; P < 0.001). There was one failure in the control group. There were no other complications in either group.

**CONCLUSION:**

Reduction of anterior shoulder dislocation using intra-articular lidocaine injection is effective, since it is safe and diminishes the pain.

**CLINICAL TRIAL REGISTRATION:**

ISRCTN27127703.

## RESUMO

**CONTEXTO E OBJETIVO:**

A luxação do ombro é a mais frequente luxação das grandes articulações. O objetivo foi comparar a efetividade da redução da luxação anterior aguda do ombro, com ou sem anestesia articular.

**TIPO DE ESTUDO E LOCAL:**

Ensaio clínico randomizado desenvolvido na Escola Paulista de Medicina - Universidade Federal de São Paulo (EPM-Unifesp).

**MÉTODOS:**

De março de 2008 a dezembro de 2009 foram recrutados 42 pacientes com luxação anterior do ombro agudas. Foi comparada a redução de tração e contra-tração com e sem anestesia intra-articular com lidocaína. Como desfecho primário, a dor foi avaliada por meio da escala visual analógica antes da redução e um e cinco minutos após a redução do ombro. Complicações também foram avaliadas.

**RESULTADOS:**

Foram incluídos 42 pacientes: 20 no grupo sem anestesia (grupo controle) e 22 no grupo que recebeu injeção intra-articular de lidocaína. O grupo que recebeu lidocaína intra-articular teve estatisticamente menos dor no primeiro (média = 2,1; 0 a 5,0, desvio padrão, DP, de 1,3, *versus* média = 4,9; 2,0 a 7,0; DP = 1,5; respectivamente; P < 0,001) e no quinto minutos (média = 1,0; 0 a 3,0; DP = 1,0 *versus* 4,0; 1,0 a 6,0; DP = 1,4; respectivamente; P < 0,001) após a redução em comparação ao grupo controle. Houve uma falha no grupo controle. Não houve complicações adicionais em ambos os grupos.

**CONCLUSÃO:**

A redução da luxação do ombro com o uso de lidocaína intra-articular é efetiva, pois é segura e proporciona menos dor.

## INTRODUCTION

Shoulder dislocation consists of total loss of joint congruence between the humeral head and the glenoid articular surface. It is the most common dislocation among the dislocations in large joints,^1^ corresponding to approximately 50% of all dislocations attended in emergency rooms.^2^ Anterior dislocations account for 96% of shoulder dislocations.^3^ Shoulder dislocation is an orthopedic emergency, and its initial treatment requires restoration of glenohumeral congruence as early as possible. Treatment can usually be carried out conservatively in the emergency room.

The ideal reduction method should be simple, fast, effective and nontraumatic, with minimal pain, and should not cause further injury to the affected shoulder.^4^ Among the several reduction techniques that have been described,^5^^,^^6^^,^^7^^,^^8^ the technique of traction and countertraction without administration of analgesic is the one most used in Brazil.^9^ However, to improve the effectiveness of the reduction maneuver, pain and muscle relaxation need to be controlled.^4^

A number of methods provide pain relief to facilitate reduction, including intravenous sedation/analgesia, anesthetic gas (nitrous oxide plus oxygen, 50% each, Entonox) and regional anesthetic techniques. Nevertheless, in standard practice within our setting, these resources are only used if reduction attempts fail.^9^

Intra-articular local lidocaine injection is a means of achieving analgesia and also provides adequate muscle relaxation. It enables a higher success rate in the reduction maneuver, with less pain, and has the advantage of allowing prompt patient discharge once reduction has been achieved.^10^^,^^11^^,^^12^ Additionally, this technique can be performed in emergency rooms, and the costs relating to its use are low.^10^^,^^11^^,^^12^

## OBJECTIVE

To evaluate the effectiveness of intra-articular lidocaine injection for closed reduction of anterior shoulder dislocations, in comparison with no analgesia.

## METHODS

This prospective randomized study was conducted between March 2008 and December 2009. The protocol number provided upon approval of the study by our institution’s Ethics Committee in March 2008 was 1019/08.

All patients with acute anterior shoulder dislocation who were treated in the Emergency Room in the study period were included in the study. The diagnostic criteria used were clinical findings, such as: shoulder deformity; acute pain and disability in relation to active and passive mobility of the shoulder; and radiographic findings showing total loss of articular congruity between the humeral head and glenoid cavity, as demonstrated by frontal, lateral and axillary shoulder radiographs.

Patients who were diagnosed with fracture-dislocation of the shoulder joint, except those with Hill-Sachs lesions, were disqualified. Patients with immature skeletons (open physis) or who underwent surgery, had previous fractures in the affected shoulder, were patients presenting contraindications for lidocaine use or refused to sign the consent form were not included in the study.

Patients were randomly allocated according to instructions contained in 54 opaque, sealed envelopes that had been sequentially numbered using a randomization program (www.randomizer.com), to one of two study groups: one group that received intra-articular injection of 20 ml of 1% lidocaine and underwent shoulder dislocation reduction by means of the traction and countertraction technique; or a second group that underwent the same reduction technique but received no analgesia or anesthesia before the reduction.

The patients who fulfilled the study inclusion and exclusion criteria each received a sequential registration number and a sealed, opaque envelope marked with the number corresponding to their registration. The envelope contained information regarding the treatment method that had been randomly assigned to the patient’s registration number. The attending physician led the patient to a treatment room where, after the door had been closed and the envelope had been opened, either an intra-articular lidocaine injection was applied or no injection was given, in accordance with the method disclosed. In both cases, the anatomical region of the arm where intra-articular anesthetic would be applied was covered with dressings in order to conceal from the physician whether the intervention had been performed or not.

Five minutes later, two other physicians were called to perform the reduction maneuver. Both of them were blind regarding which treatment the patient had received and the patient did not reveal what treatment he/she had received.

Before application of the intra-articular injection, the patient’s affected shoulder area was swabbed three times with chlorhexidine. A 20-gauge 0.7 × 40-mm needle was used to inject 20 ml of 1% lidocaine into the shoulder joint immediately distally to the lateral border of the acromion, towards the glenoid cavity.

Whether or not the patients had undergone the intervention, they were all placed supinely on a stretcher with the affected shoulder at 60^o^ abduction. The pull maneuver and countertraction were performed with a bed sheet placed under the patient’s armpit.

As the primary outcome, pain was assessed through application of a visual analogue scale (VAS) before the reduction and one and five minutes after the reduction maneuver was performed. The assessor was blind regarding whether any intervention had been performed, because of the dressings on the shoulders.

As secondary outcomes, we determined the time span required to achieve shoulder joint reduction, in minutes. Neurological, vascular and infectious complications and occurrences of failures were also assessed. Failure was defined as lack of success in performing the reduction, after a 10-minute attempt.

The sample size was determined as 20 patients per group by applying a statistical power of 90% and taking a confidence interval of 95%. The standard deviation was set as a decrease of three points down the visual analogue pain scale in the group that received the intra-articular injection of lidocaine, compared with the control group.

Pearson’s chi-square test and Fisher’s exact test^13^ were used to carry out statistical analysis to compare the groups. For the visual analogue score and the time to span to achieve shoulder reduction, analysis of variance (ANOVA) was used with a fixed-effect model. Conclusions were drawn from inferential statistical analysis at a significance level a equal to 5%.

In accordance with the intention-to-treat principle, the progress of patients whose treatment failed for some reason or who presented complications due to the reduction was monitored, and the results obtained were included in the group to which they had initially been assigned.

## RESULTS

Fifty-four patients with anterior shoulder dislocation were admitted to the emergency room over the period between June and November 2008. Six patients who presented fractures of the major tuberosity, four patients who were unconscious in the emergency room and two patients who refused to participate in the study were excluded.

**Figure 1.**
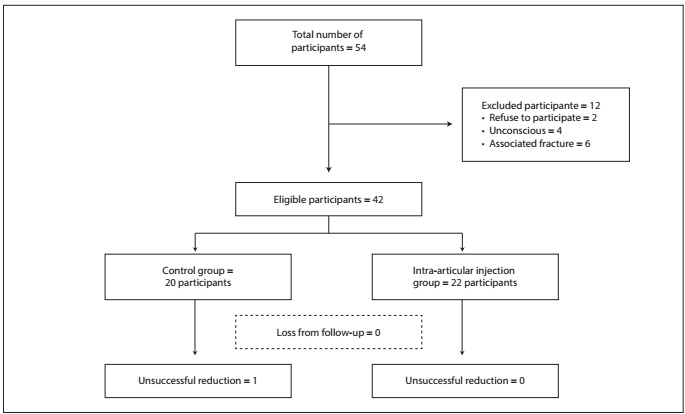
Flow chart of participants.

Forty-two patients were included: 20 were assigned to the control group, and 22 to the group that received intra-articular lidocaine injections (Figure 1).

It was observed that the two groups presented similar age, weight and time lag between dislocation and the reduction procedure. Moreover, previous dislocation and subluxation episodes were reported by both groups (Table 1).

**Table 1.** Age, weight, time lag from dislocation until the start of the reduction procedure (in minutes and hours) and previous dislocation

	Control group (n = 20)				Lidocaine group (n = 22)				P
	Mean	Min	Max	SD	Mean	Min	Max	SD	
Age (years)	32.6	18.0	78.0	14.1	39.1	19.0	74.0	16.9	0.180
Weight (kg)	70.5	46.0	100.0	12.2	72.2	48.0	90.0	10.4	0.637
Dislocation time lag (minutes)	153.6	30.0	1185.0	252.4	121.9	30.0	300.0	94.7	0.593
Dislocation time lag (hours)	2.6	0.5	19.8	4.2	2.0	0.5	5.0	1.6	0.593
Previous dislocation episodes (number)	2.3	0.0	20.0	4.5	1.5	0.0	10.0	2.7	0.521

Min = minimum; Max = maximum; SD = standard deviation.

**Figure 2.**
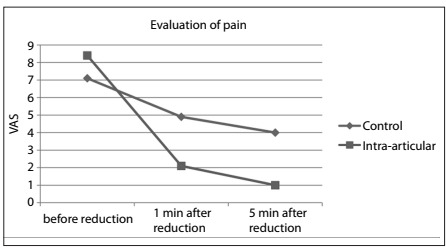
Evaluation of pain using visual analogue scale (VAS) before and after reduction.

Regarding the primary outcome, the group that received intra-articular treatment presented a statistically greater decrease in pain over time lower than what was shown by the control group, in both the first and fifth minutes (P < 0.001) (Figure 2, Table 2).

**Table 2.** Pain comparison between the groups

	Control group				Lidocaine group (n = 22)				P*
	Mean	Min	Max	SD	Mean	Min	Max	SD	
VAS score before reduction	7.1	4.0	10.0	1.8	8.4	5.0	10.0	1.5	0.012
VAS score one minute after reduction	4.9	2.0	7.0	1.5	2.1	0.0	5.0	1.3	< 0.001
VAS score five minutes after reduction	4.0	1.0	6.0	1.4	1.0	0.0	3.0	1.0	< 0.001

VAS = visual analogue scale; Min = minimum; Max = maximum; SD = standard deviation. ^*^Repeated-measurement analysis of variance (ANOVA).

Regarding secondary outcomes, the time taken to achieve the reduction was shorter in the group treated with intra-articular analgesic (P = 0.005) (Table 3).

**Table 3.** Time taken to achieve shoulder reduction in the groups

	Control group (n = 20)				Lidocaine group (n = 22)				P*
	Mean	Min	Max	SD	Mean	Min	Max	SD	
Time taken for reduction (minutes)	4.9	0.5	15.0	3.8	2.0	0.3	8.0	2.1	0.005

Min = minimum; Max = maximum; SD = standard deviation. ^*^Analysis of variance (ANOVA).

There was one failure in the control group. This patient was taken to the operating room, and the shoulder joint was successfully reduced by surgical means. There were no other complications in either group.

There was no loss during the follow-up, among the patients in both groups.

## DISCUSSION

Shoulder dislocation is a common condition in medical practice.^14^ Some authors have stated that no form of analgesic or sedation is required for reduction of anterior shoulder dislocation.^10^^,^^15^^,^^16^^,^^17^ These authors have maintained that such procedures could lead to complications like respiratory depression and seizures, which would therefore require cardiorespiratory monitoring following administration of these substances.^18^ This would add time and cost to the procedure, as well as entailing reliance on other physicians and emergency staff, to assist with the reduction.^16^ These claims explain why, in Brazil, most emergency physicians do not use any type of analgesic or sedation.^9^

One alternative described in the literature is the intra-articular lidocaine injection technique, and some authors have suggested that this would be an excellent choice for improved analgesia, in addition to its low costs and low complication rates.^10^^,^^11^^,^^12^^,^^19^ Despite citations in the literature of success rates of between 60% and 90% for reduction techniques using different maneuvers,^20^^,^^21^^,^^22^^,^^23^^,^^24^^,^^25^^,^^26^ our aim here was to test the effectiveness of this technique in combination with intra-articular injection, according to the hypothesis that this combination could produce faster and less painful treatment for shoulder dislocation.

The primary outcome with regard to articular anesthetic injection showed it provided lower pain levels, as assessed by a visual analogue scale, at both the first and fifth minutes after reduction. This result is consistent with other studies that compared the use of intra-articular injection methods with intravenous analgesics and sedatives.^10^^,^^11^^,^^12^^,^^27^ The time required to achieve the reduction was also shorter in the intra-articular injection group, thus supporting the hypothesis that improved analgesia secondarily provides greater muscle relaxation, which facilitates shoulder reduction.^10^

One patient was withdrawn from the control group, whereas in the group treated with intra-articular lidocaine injection, all the individuals remained until the end of the study. One previous study^10^ found similar results with regard to reduction failures; however, others have reported that failures also occurred using the intra-articular injection method. The latter may be due to the number of patients evaluated.^11^^,^^12^

In general, sedation with analgesia is not routinely used in Brazil, probably due to hospital issues relating to post-reduction observation and monitoring. Thus, the reduction method using lidocaine injection is a good alternative for treating shoulder dislocations, providing more effective reduction, faster patient discharge and lower pain levels.

### Implications for practice

Reduction of anterior shoulder dislocation using intra-articular lidocaine injection provides a lower pain level than observed in reduction without anesthesia. It seems to be safe and should be used in clinical practice.

### Implications for future research

Further research exploring the safety of various types of reductions, making comparisons between them, and trials with large numbers of participants are justified.

## CONCLUSION

Intra-articular lidocaine for anterior shoulder dislocation treatment reduces pain, in comparison with the same method without analgesia.
